# Effect of Air Pollution on Glutathione S-Transferase Activity and Total Antioxidant Capacity: Cross Sectional Study in Kuwait

**DOI:** 10.5696/2156-9614-10.27.200906

**Published:** 2020-08-25

**Authors:** Abeer M. Almutairi, Yazan Akkam, Mohammad F. Alajmi, Nosaibah Akkam

**Affiliations:** 1 Science Department, College of Basic Education, Public Authority for Applied Education and Training, (PAAET), Alardyia, Kuwait; 2,4 Department of Medicinal Chemistry and Pharmacognosy, Faculty of Pharmacy, Yarmouk University, Irbid, Jordan; 3 Department of Mathematics and Natural Sciences, College of Arts and Sciences, Gulf University for Science and Technology, Mubarak Al-Abdullah, Kuwait

**Keywords:** glutathione S-transferase, total antioxidant, 1-chloro-2, 4-dinitrobenzene, air-pollution, oxidative stress

## Abstract

**Background.:**

Air pollution poses a significant threat to human health worldwide. Investigating potential health impacts is essential to the development of regulations and legislation to minimize health risks.

**Objectives.:**

The aim of the present study was to investigate the potentially hazardous effect of air pollution on the Ali Sabah Al Salem residential area in Kuwait by comparing the pollution level to a control area (Al-Qirawan) by assessing two biomarkers: erythrocyte glutathione S-transferases (e-GST) and total blood antioxidant, and then correlating the activity to pollution-related oxidative stress.

**Methods.:**

The average concentrations of several airborne gases were measured at Ali Sabah Al Salem and Al-Qirawan, including ozone, carbon monoxide, nitrogen dioxide, nitrogen oxides, particulate matter less than 10 μm (PM_10_), sulfur dioxide, ammonia, carbon dioxide, hydrogen sulfide, methane, and non-methane hydrocarbon. A total of fifty-eight participants were sampled from two different areas and divided into two groups. The study group was composed of 40 residents exposed to polluted ambient air in the Ali Sabah Al Salem residential area. A reference group composed of 18 residents in the Al-Qairawan area living far from major pollution sources was also tested.

**Results.:**

All measured gases were higher in concentration at Ali Sabah Al Salem compared to the Al-Qirawan area. Furthermore, PM_10_ and sulfur dioxide were higher than World Health Organization (WHO) guidelines. The e-GST activity was lower among participants of the Ali Sabah Al Salem residential area compared to participants living in the Al-Qairawan area. The total antioxidant capacity in whole blood of Ali Sabah Al Salem residents was significantly (p<0.0001) higher than in control subjects.

**Conclusions.:**

Residents in Ali Sabah Al Salem are exposed to a high level of air pollution that has a serious impact on glutathione S-transferases levels. Subsequently, regulations on pollution sources are needed to lower current health risks. Furthermore, the present study provides evidence that finger-prick blood sampling is a quick, non-invasive method suitable for screening e-GST activity and total antioxidants which may be applied for surveillance purposes.

**Participant Consent.:**

Obtained

**Ethics Approval.:**

The study was approved by the Scientific Research Committee of the Public Authority for Applied Education and Training, Kuwait.

**Competing Interests.:**

The authors declare no competing financial interests.

## Introduction

Air pollution has become a major problem in recent decades and has been linked to serious impacts on human health and the environment.^1^,^2^ Therefore, it is essential to develop early warning biomarkers that reflect adverse biological responses towards pollutants. A biomarker is defined as “changes in a biological response (molecular, cellular, physiological, behavioral) due to exposure to pollutants.”^3^

Glutathione S-transferases (GST) are enzymes that protect organisms from endogenous and exogenous toxic compounds.^4^ Their defensive activity is attained by the covalent linkage of glutathione (GSH) to xenobiotic compounds, yielding a GSH conjugate; which is less toxic than the parent compound.^5^ Three different isoforms of GST are found in humans: alpha, pi, and mu.^6^ In erythrocytes, the isoenzyme, GSTP1-1, represents a sensitive biomarker that has been overexpressed in case of increased blood toxicity.^7^,^8^ Theoretically in a healthy human, the level of erythrocyte glutathione S-transferases (e-GST) remains largely constant throughout life.^9^ The activity of e-GST has been correlated to several types of human diseases such as hyperbilirubinemia and uremia, in which e-GST levels are overexpressed within the body.^10^ Naturally, GSTs are found in large quantities in blood cells and plasma; therefore, ensuring sample collection and preservation for blood or plasma is extremely critical.

The most commonly used methods for screening e-GST activity and total antioxidants are finger pricking and venous blood sampling, and each method has its characteristics and advantages. Finger pricking is the preferred method for blood sampling since the procedure is relatively painless, efficient, and simple. The present study was conducted to investigate the potentially hazardous effects of air pollution on the Ali Sabah Al Salem residential area by assessing e-GST as a biomarker and investigating oxidative stress via measuring blood total antioxidants.

**Abbreviations**
*e-GST*: Erythrocyte glutathione S-transferases
*gHb*: Gram of hemoglobin
*GSH*: Glutathione
*GST*: Glutathione S-transferases
*H_2_S*: Hydrogen sulfide
*NMHC*: Non-methane hydrocarbons
*PM_10_*: Particulate Matter less than 10 μm in diameter
*SO_2_*: Sulfur dioxide
*U*: Enzyme units
*USEPA*: United States Environmental Protection Agency
*WHO*: World Health Organization

## Methods

**Figure 1:**
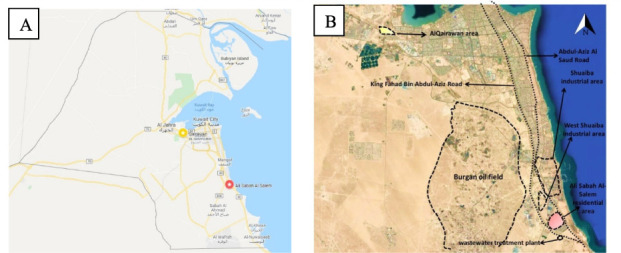
Geographic location of the study area. A) Map of Kuwait showing Al-Qairawan area (yellow shade), and Ali Sabah Al Salem residential area (red shade). B) Detailed map showing Al-Qairawan area (yellow shade), and Ali Sabah Al Salem residential area (red shade) along with possible sources of pollution.

Two distinct areas in Kuwait were selected for the present study: Al-Qairawan and Ali Sabah Al Salem. Al-Qairawan—West of Kuwait City— is assigned as a control area as it is far from any possible industrial activities or known pollution. The Ali Sabah Al Salem region—south of Kuwait City—is a suburban industrial area with an estimated population of 48 000 in 2012 *(Figure 1)*.^11^ The Al-Qairawan area is located to the west of Kuwait City with a population of approximately 25 000 according to the public authority for civil information website.^12^ Air pollution in Ali Sabah Al Salem is primarily due to its proximity to two major highways, several state-owned and private industries, and it is adjacent to the largest oilfield in the country (the Burgan) and surrounded by two large industrial areas, West and North Shuaibah industrial area, which is comprised of more than 100 private industrial companies and workshops *(Figure 1).*^13^

### Study design and participants

During the summer (April–July 2017), samples from healthy volunteers living in two areas of Kuwait, Ali Sabah Al Salem (residential) and Al-Qairawan (control area) were collected. All the volunteers were men who worked at supermarkets in the designated area for at least six months. Eighteen and 40 healthy participants were sampled from Al-Qairawan and Ali Sabah Al Salem residential areas, respectively. Participants with a history of hypertension, hyperlipidemia, heart disease, liver disease, kidney disease, diabetes, as well as smokers, were excluded *(Supplemental Material 1).* All volunteers gave their informed consent before participating in the study. The study was conducted per the Declaration of Helsinki and was approved by the Scientific Research Committee of the Public Authority for Applied Education and Training, Kuwait.

### Measuring air pollution

Pollutant concentrations were measured and recorded by air quality monitoring stations run by the Kuwait Environment Public Authority located in the selected areas. The monitoring stations measure wind speed, direction, other weather parameters, and the concentration of several air pollutants. The measurement range and detection principles of these stations have been previously described.^11^ The stations survey air pollution daily. Due to governmental regulations, air pollutant data over a period of 8 months (Jan–Aug 2017) only was accessed. Measured gases were ammonia, carbon dioxide, carbon monoxide, hydrogen sulfide, methane, nitrogen dioxide, nitrogen oxides, non-methane hydrocarbon, ozone, particle matter less than 10 μm (PM_10_) and sulfur dioxide.

### Finger-prick blood sampling

A previous study found that measuring e-GST activity either by taking a blood sample from the vein or the tip of the finger will provide the same result, and therefore, the finger prick method was used in the present study.^14^ For the finger-prick sampling, one skin puncture was made on the tip of the left ring finger using a blood sampling needle (Accu-Chek Safe-T-Pro Plus Lancets, Roche Diagnostics, USA). The first and second drops of blood produced after gentle squeezing were discarded, and then an aliquot (250 μl) of blood was taken in a ethylenediaminetetraacetic acid 0.5 ml micro-container tube, using an Innovac Quick-Draw device (Innovative Med Tech USA) (the larger volume collected decreases finger-prick drop-to-drop variability). The aliquot of blood was immediately frozen in dry ice and stored in a −80°C freezer. After thawing the blood in a 25°C water bath, 10 μl was transferred to a microtube containing 490 μl of ice-cold HPLC water. Then the mixture was vortexed and kept at −80°C for 10 minutes (the deep freezing of the lysate sample will break all the erythrocyte cells open). The erythrocyte lysate was then analyzed within one week after recovery.

### Erythrocyte glutathione S-transferases activity assay

Erythrocyte glutathione S-transferases activity was assayed spectrophotometrically with a microplate reader (bio-Tek ELx800, USA) at 25°C using 1-chloro-2,4-dinitrobenzene as a substrate. The lysate samples were removed from the −80°C freezer and allowed to thaw in a 25°C water bath. Then, 1-chloro-2,4-dinitrobenzene (100 mM at 4°C) and GSH (100 mM at −20°C) were allowed to thaw at room temperature. Each well in the microplate (Greiner Bio one, No. 655101 96) had a final volume of 200 μl. An assay cocktail was made from 197 μl of 100 mM phosphate buffer saline pH 6.5, 1 μl of 100 mM GSH, and 2 μl of 100 mM 1-chloro-2,4-dinitrobenzene. For blanks, 200 μl of the assay cocktail was used, while for each tested sample, 180 μl of the assay cocktail was used and 20 μl of the lysate sample was added to it, mixed, and after a lag time of 1 minute, after which the absorbance was measured at 340 nm every minute for 10 minutes. Each assay was performed in duplicate, and the results were repeated at least 4 times. The hemoglobin concentration was measured for each lysate sample as described previously with slight modifications using a Hemocue HB 201+ device (HemoCue America, USA). The e-GST activity was stated as enzyme units (U)/gram of hemoglobin (gHb).^15^ Glutathione unit represents the amount of enzyme that catalyzes the conjugation of 1 μmol of GSH to 1-chloro-2,4-dinitrobenzene in 1 minute at 25ºC. Furthermore, the activity of e-GST is in a linear relationship to its expression.^16^ Therefore, the increase of e-GST activity can be interpreted as a high expression of e-GST.

### Total antioxidant capacity

The total antioxidant activities in blood were measured by the iodine starch agar method as described previously.^17^ Briefly, 1.5 g agar in 100 mL water was heated for 3 minutes at 100°C. Then, the solution was cooled down to 55°C before it was mixed with iodine (6 mL of 0.005 mol/L) and starch (0.6 mL of 5 g/L) solutions. The mixture was poured in an 8 cm petri dish, with a thickness of 0.18 cm. After solidification, a 6 mm hole was created at the center of the plate. Thirty (30) μL (1:50) blood sample was incubated at the apertures. Afterward, a layer of liquid paraffin (8 mL) was added to cover the surface. The plate was then incubated (in the dark) at 4°C for 10 days. Finally, the diameter of the colorless circle was measured. The area of the reaction was calculated using Equation 1:

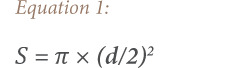
where S stands for the area of blood diffusion and *d* for the diameter of the colorless circle.

### Statistical analysis

The means and SD were calculated. Data were expressed as mean ± SD. Statistical analysis was performed using GraphPad Prism 7 software. Student's t-test was used for statistical analysis. The p-values of less than 0.05 were considered statistically significant.

## Results

The accessibility to air quality monitoring stations data was limited due to governmental regulation, only data for the period (April–August 2017) were released, which includes the sample collection. The average concentrations of measured airborne gases in both areas, as well as the World Health Organization (WHO) air quality guideline values, are summarized in Table 1. Detailed graphical representations gas concentrations over the period of sample collection are provided in Supplemental Material 2. Measurements were taken daily.

**Table 1:** Average Concentration of Air Pollutants at Ali Sabah Al Salem and Al-Qairawan Area Over a Period of 4 Months (April 1–August 1, 2017)

**Air pollutant**	**Ali Sabah Al Salem (concentration ± SD)**	**Al-Qairawan (concentration ± SD)**	**WHO air quality guideline values**^17^
Ozone	0.0252 ± 0.009 ppm	0.0223 ± 0.00228 ppm	0.050 ppm (annual mean)
Carbon monoxide	0.699 ± 0.17 ppm	0.492 ± 0.09 ppm	35 ppm (not to be exceeded more than once per year) ^*^
Nitrogen dioxide	0.0319 ±0.009 ppm	0.0279 ± 0.0022 ppm	0.053 ppm (annual mean)
Nitrogen oxides	0.0458 ±0.015 ppm	0.03522 ±0.00156 ppm	0.01–0.05 ppm
Particulate matter - less than 10 pm	114.94 ± 13.19 μg/m^3^	24.46 ± 5.65 μg/m^3^	20 μg/m^3^ (annual mean; not to exceed 150 μg/m^3^ more than once per year on average over 3 years)^*^
Sulfur dioxide	0.01 ±0.0049 ppm	0.003 ± 0.001 ppm	0.007 ppm (24-hour mean; not to exceed 0.05 ppm more than once per year)^*^
Ammonia	0.017 ±0.008 ppm	NA	NA
Carbon dioxide	388.27 ± 12.5 ppm	366.256 ± 20.5 ppm	250–350 ppm
Hydrogen sulfide	0.0092 ± 0.003 ppm	NA	0.000267 ppm^**^
Methane	1.810 ±0.199 ppm	NA	1.824 ppm^**^
Non-methane hydrocarbon	0.581 ±0.17 ppm	NA	NA

Abbreviation: NA, not applicable.

* United States Environmental Protection Agency regulations.

^**^ Average ambient air level as a result of natural sources.

#### Sulfur dioxide

**Figure 2:**
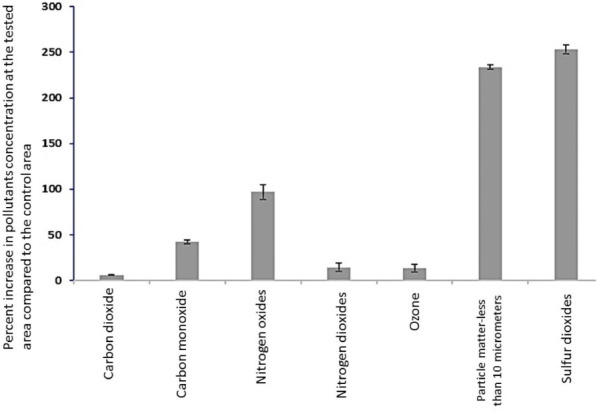
The increase of air pollution in Ali Sabah Al Salem over Al-Qairawan

The results showed that the average concentrations of sulfur dioxide (SO_2_) at Ali Sabah Al Salem and Al-Qairawan were 0.01 ± 0.0049 ppm and 0.003 ± 0.001 ppm, respectively. According to *Table 1*, Ali Sabah Al Salem reading (0.01) is exceeding the WHO limit (0.007), and more than three-fold higher than in Al-Qairawan *(Figure 2).* Detailed daily measurements are presented in Supplemental Material 2, Figure 1.

#### Particulate matter—less than 10 μm

The results showed that the average concentrations of PM_10_ at Ali Sabah Al Salem and Al-Qairawan were 114.94 ± 13.19 μg/m^3^ and 24.46 ± 5.65 μg/m^3^, respectively. Both readings were higher than 20 μg/m^3^, which is the WHO reference value *(Table 1).* However, the concentration in Ali Sabah Al Salem was more than 200% higher than in Al-Qairawan *(Figure 2).* Detailed daily measurements are shown in Supplemental Material 2, Figure 2.

#### Ozone

The results showed that the average concentrations of ozone in Ali Sabah Al Salem and Al-Qairawan were 0.0252 ± 0.009 ppm and 0.0223 ± 0.00228 ppm, respectively. Both readings were less than 0.05 ppm, which is the WHO reference value *(Table 1).* However, the concentration in Ali Sabah Al Salem was 13% higher than in Al-Qairawan *(Figure 2).* Detailed daily measurements are shown in Supplemental Material 2, Figure 3.

#### Nitrogen oxides

The results showed that the average concentrations of nitrogen oxides in Ali Sabah Al Salem and Al-Qairawan were 0.0458 ± 0.015 ppm and 0.03522 ± 0.00156 ppm, respectively. Both readings fit into the acceptable range of the WHO (0.01–0.05 ppm) *(Table 1).* However, the concentration in Ali Sabah Al Salem was one-fold higher than in Al-Qairawan *(Figure 2)*. Detailed daily measurements are presented in Supplemental Material 2, Figure 4.

#### Nitrogen dioxide

The results showed that the average concentration of nitrogen dioxide at Ali Sabah Al Salem and Al-Qairawan weres 0.0319 ± 0.009 ppm and 0.0279 ± 0.0022 ppm, respectively. Both readings were less than 0.053 ppm, which is the WHO reference value *(Table 1).* However, the concentration in Ali Sabah Al Salem was 15% higher than in Al-Qairawan *(Figure 2).* Detailed daily measurements are presented in Supplemental Material 2, Figure 5.

#### Carbon monoxide

The results showed that the average concentrations of carbon monoxide at Ali Sabah Al Salem and Al-Qairawan were 0.699 ± 0.17 ppm and 0.492 ± 0.09 ppm, respectively. Both readings were within the acceptable range of the United States Environmental Protection Agency (USEPA) of 35 ppm *(Table 1).* However, the concentration in Ali Sabah Al Salem was 42% higher than in Al-Qairawan *(Figure 2).* Detailed daily measurements are shown in Supplemental Material 2, Figure 6.

#### Carbon dioxide

The results showed that the average concentrations of carbon dioxide in Ali Sabah Al Salem and Al-Qairawan were 388.27 ± 12.5 ppm and 366.256 ± 20.5 ppm, respectively. Both readings were on the edge of the normal range (350 ppm) *(Table 1).* However, the concentration in Ali Sabah Al Salem was 5% higher than in Al-Qairawan *(Figure 2).* Detailed daily measurements are shown in Supplemental Material 2, Figure 7.

#### Ammonia

The results showed that the average concentrations of ammonia at Ali Sabah Al Salem were 0.017 ± 0.008 ppm. The air quality monitoring stations at Al-Qairawan did not have ammonia sensors. Ammonia is a normal component of the ambient air, and according to USEPA and WHO regulations *(Table 1),* there is no normal range for ammonia. Detailed daily measurements are presented in Supplemental Material 2, Figure 8.

#### Hydrogen sulfide

The results showed that the average concentrations of hydrogen sulfide (H_2_S) at Ali Sabah Al Salem were 0.0092 ± 0.003 ppm. The air quality monitoring stations at Al-Qairawan did not have H_2_S sensors. The concentration at Ali Sabah Al Salem was higher than the natural concentration of H_2_S (0.000267 ppm) *(Table 1).* Detailed daily measurements are presented in Supplemental Material 2, Figure 9.

#### Methane

The results showed that the average concentration of methane in Ali Sabah Al Salem was 1.810 ± 0.199 ppm. The air quality monitoring stations at Al-Qairawan did not have methane sensors. The concentration at Ali Sabah Al Salem was on the edge of the background concentration *(Table 1).* Detailed daily measurement was shown in Supplemental Material 2, Figure 10.

#### Non-methane hydrocarbon

The results showed that the average concentration of non-methane hydrocarbons (NMHC) at Ali Sabah Al Salem was 0.581 ± 0.17 ppm. The air quality monitoring stations at Al-Qairawan did not have NMHC sensors *(Table 1).* Detailed daily measurements are presented in Supplemental Material 2, Figure 11.

### Effect of air pollution on e-GST activity

**Figure 3:**
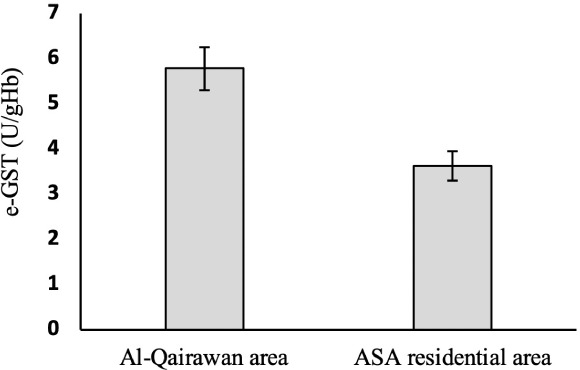
Comparison of e-GST activity between Ali Sabah Al Salem and Al-Qairawan. Error bars represent the standard deviation.

Statistical *t*-test showed that the mean e-GST enzyme activity of Ali Sabah Al Salem residents of (3.6 ± 0.3 U/(gHb)) was significantly lower than the residents of Al-Qairawan (5.8 ± 0.5 U/(gHb)). All the data are summarized in Table 2 and shown in Figure 3.

**Table 2:** Subject Age, Concentrations of Hemoglobin, and e-GST Activity by Subject Group

**Area of the subjects**	**Al-Qairawan**	**Ali Sabah Al Salem residential area**	***p*-value for difference among groups**
Number	18	40	
Age (years)^*^	(37 ± 8)	(37 ± 9)	
Hemoglobin (mg/ml)^*^	(44.4 ± 4)	(47 ±3.4)	
e-GST activity (units/(gHb)	(5.8 ±0.5)	(3.6 ±0.3)	<0.0001

^*^mean ± standard deviation

### Effect of air pollution on total antioxidant capacity

As showed in Table 3, the average diffusion area at Ali Sabah Al Salem samples was 4.29 ± 0.18 cm^2^, and 3.11 ± 0.1 cm^2^ in Al-Qairawan samples. The diffusion area is proportional to antioxidant activity in the blood; the larger the diffusion area, the greater the antioxidant activities. Therefore, more antioxidant activity was measured in Ali Sabah Al Salem samples compared to Al-Qairawan at *p* < 0.05 *(Table 3).*

**Table 3:** Blood Diffusion Area of Healthy Subjects in Ali Sabah Al Salem and Al-Qairawan According to the Iodine Starch Agar Method

**Method**	**Area**	**Diffusion area (cm^2^, mean ± SD)**	***t***	***P***
Iodine	Al-Qairawan	3.11 ±0.1	−32.103	< 0.0001
	Ali Sabah Al-Salem	4.29 ±0.18		

## Discussion

Air pollution has gained significant attention in developing countries (including Kuwait), with industrial activities reported as the primary sources.^11^,^18^ Air pollution has been associated with oxidative stress and is a major contributing factor to chronic diseases, including cardiovascular and pulmonary diseases.^19^,^20^ Increased exposure to pollutants has created a critical need for biological markers to detect oxidative stress. Byproducts of oxidative stress in air pollutants may serve as initiators and promoters of the damages that induce chronic diseases.^20^ Pollution may augment oxidative stress not only through the production of reactive oxygen species but also through the weakening of antioxidant defense mechanisms.^15^,^16^ It has been reported that reactive oxygen species may overcome the capacity of the oxidant defense system.^16^

Oxidative damage is normally tolerated by a combination of biological antioxidant systems including enzymatic and non-enzymatic reactions. Glutathiones, members of the antioxidant system, are important detoxification enzymes.^21^ Furthermore, GST is responsible for the high-capacity metabolic inactivation of electrophilic compounds.^22^ Hence, the glutathione redox status is critical for various biological events.^23^ An increase in GST enzymatic activity would be expected in response to exposure to electrophiles or oxidizing agents.^24^

Among GSTs classes, blood GSTs have been the main emphasis, they are thought to be subject to a high internal rate of reactive oxygen species produced from hemoglobin auto-oxidation.^25^ Blood is the first compartment to be exposed to oxidative stress, including red blood cells. Furthermore, erythrocytes are subjected to high risk of oxidative damage due to the exposure of high oxygen tension, and lack of damage repair mechanisms.^4^ Consequently, erythrocytes rely completely on the antioxidant defensive components throughout their life span.^26^ Therefore, the effect of pollution on the induction of oxidative stress may be monitored or measured by studying blood GST, which includes serum GST and erythrocyte GST.

Recently, several types of air pollutants have been detected in the region: gaseous pollutants (SO_2_, nitrogen dioxide, H_2_S, ozone, carbon monoxide), organic pollutant compounds (methane, ammonia, methylamide), and particulate matter.^11^ According to the WHO and the USEPA, most of these pollutants have been linked to oxidative stress and serious health concerns. Al-Qairawan does not have any type of industrial activity or any known source of air pollution.

According to WHO and the USEPA, only ozone, carbon monoxide, nitrogen dioxide, particulate matter, and SO_2_ are subject to National Ambient Air Quality Standards (NAAQS) .^27^ However, other gases measured in this study may also be hazardous air pollutants according to the USEPA, but this depends on their concentration, hence no normal range was indicated.

According to the data, ozone, carbon monoxide, and nitrogen dioxide were in acceptable ranges in both areas compared to USEPA and WHO standards *(Table 1).* On the other hand, the concentrations of PM_10_ and SO_2_ were higher than the standards in the Ali Sabah Al Salem area. For PM_10_, both readings were higher (Ali Sabah Al Salem and Al-Qairawan) than the WHO limit. In general, the concentration of all measured gases was higher in Ali Sabah Al Salem than Al-Qairawan *(Figure 2).* Some of these airborne gases were not measured in the Al-Qairawan area, such as ammonia, hydrogen sulfide, methane, and non-methane hydrocarbon. The government did not add detectors for those gases in the Al-Qairawan area as there were no contamination sources nearby.

Sulfur dioxide is generated from the burning of sulfur-containing fossil fuels (coal and oil) for domestic heating, power generation, and motor vehicles. As illustrated in Supplemental Material 2, Figure 1, the concentration of SO_2_ was twofold higher at Ali Sabah Al Salem compared to Al-Qairawan during the surveillance period. According to WHO air quality guidelines, the concentration of SO_2_ should be less than 20 μg/m^3^ or (0.007 ppm) on a 24-hour average.^27^ The average SO_2_ in Ali Sabah Al Salem was 0.01 ± 0.0049 ppm, which exceeded the WHO guideline. In Al-Qairawan, the concentration was within the guideline range (0.003 ± 0.001 ppm). It has been reported that exposure to SO_2_ enhances oxidative stress and lipid peroxidation, and consequently, leads to inflammation of the respiratory tract causing coughing, mucus secretion, asthma and chronic bronchitis.^28^–^31^

Particulate matter less than 10 μm is comprised of dust particles with an aerodynamic diameter smaller than 10 μm. Kuwait is a semi-desert dryland, and sandstorms often occur, especially in summer.^11^ The results show that the concentration of PM_10_ in Ali Sabah Al Salem (114.94 ± 13.19 μg/m^3^) was greater than the WHO 24-hour guideline (20 μg/m^3^) *(Table 1*). On the other hand, the concentration at Al-Qairawan was close to the guidelines with a concentration of 24.46 ± 5.65 μg/m^3^. The increase in PM_10_ was more than 200% greater in Ali Sabah Al Salem than Al-Qairawan *(Figure 2),* and WHO guidelines. Furthermore, according to the USEPA National Ambient Air Quality Standards, PM_10_ should not exceed more than 150 μg/m^3^ once per year on a 24 hour-average.^32^ The surveillance data *(Supplemental Material 2, Figure 2),* shows that PM_10_ exceeded the daily concentration 19 times over 4 months, and measured as high as 900 μg/m^3^ on one day. Particulate matter less than 10 μm has been associated with several health problems and is considered a potential cause of oxidative stress and as a free radical.^33^–^41^

Despite the fact that the ozone annual mean was within the WHO accepted range in the Ali Sabah Al Salem area, the daily records surpassed the average 13 times (greater than 0.05 ppm) *(Supplemental Material 2, Figure 3),* and recorded a 300% increase in a single day, suggesting the possibility of acute effects. Ozone comes from chemical reactions between oxides of nitrogen and volatile organic compounds in the presence of heat and sunlight. Both pollutants come from vehicular traffic, power plants, industrial boilers, refineries and chemical plants.^42^ The Ali Sabah Al Salem area is heavily occupied with refineries and industrial factories which enhances the emission of ground ozone precursors. Moreover, Kuwait's climate is sunny and hot throughout the year, which facilitates the production of ground ozone.^43^ Despite these circumstances that point toward the existence of a high level of ground ozone, official data showed it was within the acceptable range.

The most common sources of H_2_S emissions are oil and natural gas extraction and processing. The average ambient air level as a result of natural sources is 0.4 μg/m^3^ or (0.0002 ppm).^44^ The concentration in Ali Sabah Al Salem was higher than the accepted range (0.0092 ± 0.003 ppm) *(Table 1).* Furthermore, the concentration was 30-fold higher than the normal range. Hydrogen sulfide in the air is commonly oxidized by molecular oxygen and hydroxyl radicals, forming the sulfhydryl radical and ultimately sulfur dioxide that will pile into the SO_2_ ambient air concentration. It has been reported that exposure to excessive atmospheric H_2_S induces inflammation, oxidative stress, energy metabolism dysfunction, and adverse health effects.^45^,^46^

According to the World Meteorological Organization, there are five long-lived greenhouse gases: carbon dioxide, methane, nitrous oxide, dichlorodifluoromethane, and trichlorofluoromethane. The toxicity of carbon dioxide is related to concentration. More than 70% of the emitted carbon dioxide comes from fossil fuel combustion.^47^ According to the standard range, the background (normal) outdoor air level can reach up to 300 ppm of carbon dioxide with no adverse effects.^48^ Adverse effects start at a concentration of 1000 ppm.^48^ Despite the fact that Kuwait was considered the third world's largest per capita carbon dioxide emitter in 2017 at 25 tons per person, the recorded carbon dioxide of both areas is considered to be on the borderline of the normal range.^49^ However, the concentration of carbon dioxide in Ali Sabah Al Salem was higher than Al-Qairawan *(Figure 2, Table 1).*

Unfortunately, ammonia is, so far, not included as a species of routine monitoring in the NAAQS. Moreover, only a few measurements and studies on atmospheric ammonia have been reported. According to previous studies, the concentration of 0.017 ppm atmospheric ammonia recorded in Ali Sabah Al Salem is higher than those recorded in urban locations of Shanghai, Bejing, Seoul, Barcelona, and Atlanta, Georgia, Houston, Texas, Kent, Washington, and Wisconsin^50^. Ammonia pollution is mainly due to the chemical industry or agricultural-related fertilizer application, livestock waste, compost, and water treatment plants. It has been reported that exposure to a high level of ammonia may induce oxidative stress.^51^,^52^

The concentration of methane, a greenhouse gas, in Ali Sabah Al Salem was within the background level. The concentration was 1.810 ± 0.199 ppm and the background level was 1.824 ppm.^53^ Non-methane hydrocarbons are volatile organic compounds. Non-methane hydrocarbons contain a large number of organic compounds such as ethane, acetylene, ethylene, isopentane, benzene, toluene, and isoprene. Some of these compounds are carcinogenic compounds such as benzene and 1.3-butadiene.^54^ Non-methane hydrocarbons may also have an indirect effect on ozone production and secondary organic aerosol formation. Furthermore, it can undergo photochemical reactions and be oxidized forming secondary volatile organic compounds which can lead to the formation of many oxidants including ozone.^18^ Unfortunately, NMHC data was too incomplete to conclude, as the NMHC members were unidentified and their concentration was unknown.

The results of the present study demonstrate that the concentration of airborne gasses was higher in Ali Sabah Al Salem than in Al-Qairawan, and SO_2_ and PM_10_ were higher than the WHO guideline values. Previous studies have reported that the levels of pollutants in the Ali Sabah Al Salem area were very high and exceeded the Kuwait Environmental Public Authority standards.^11^ As previously mentioned, air pollutants in Ali Sabah Al Salem (PM_10_, SO_2_, NMHC, H_2_S) and airborne gas ammonia can enhance oxidative stress and initiate the production of reactive oxygen species. Hence, there is a significant difference in e-GST activity in healthy subjects living in the Ali Sabah Al Salem residential area compared to those living in Al-Qairawan.

Even though the Ali Sabah Al Salem area is exposed to air pollution to a greater degree, the results show that e-GST activity is lower than Al-Qairawan. Two possible mechanisms may explain this result. The first mechanism is the consumption of antioxidants.^55^ Each air pollutant detected in the Ali Sabah Al Salem area is itself a potent oxidizing agent.^56^ For instance, ozone is known to initiate intracellular oxidative stress via ozonide and hydroperoxide formation.^19^ Therefore, the existence of such pollutants will lower antioxidant activity in the human body and overcome the regeneration of e-GST. It has been reported that workers exposed to 1,3-butadiene (an oxidizing compound) in the air of an industrial area displayed lower e-GST activity.^24^

The second possible mechanism involves the direct inhibition of GSH synthesis and activities of superoxide dismutase and GST.^12^,^57^ It has been reported that oxidative stress derived from an imbalance between reactive oxygen species and individual antioxidant activity may lead to the damaging of macromolecules such as DNA and ribonucleic acid including genes responsible for the GST enzyme.^58^ Moreover, it has been suggested that air pollutants may cause mutations in GST genes.^59^

According to the results, residents of Ali Sabah Al Salem may lack cellular protection provided by e-GST. Thus, this population has been exposed to high concentrations of oxidizing toxins to a limit beyond the capacity of the oxidant defense system. Hence, the oxidative damage may encounter proteins, lipids, and DNA, and subsequently alter the expression of some xenobiotic-metabolizing enzymes and antioxidant proteins.^60^ Similar to the results of our study, it has been reported that the exposure to an oxidizing agent (smoking) lowers erythrocyte selenium-dependent glutathione peroxidase activities compared to non-smokers.^12^ Comparably, the reduction in e-GST activity in the present study (3.6 U/gHb) was similar to the effect of smoking hookah (3.7 U/gHb).^12^

Glutathione is not the only enzyme induced to counteract reactive oxygen species formation. A series of antioxidants and detoxification enzymes can be initiated, such as superoxide dismutase, catalase, glutathione peroxidase, thioredoxin, and peroxiredoxin.^61^ Therefore, antioxidant activity in blood is not limited to GST. Humans have a highly complex antioxidant protection system with multiple components; endogenous antioxidants (such as bilirubin, lipoic acid, ubiquinone), dietary antioxidants (such as vitamin E, vitamin C, beta carotene, flavonoids) and metal-binding proteins (albumin, ferritin, transferrin).^20^,^62^ This is demonstrated in the results, as the total antioxidant activity was higher in Ali Sabah Al Salem compared to Al-Qairawan. Hence, the human body successfully compensates for a low level of GST, observed in Ali Sabah Al Salem, by inducing other antioxidant enzymes and mechanisms. These results also confirm that residents of Ali Sabah Al Salem have been exposed to oxidative stress, primarily due to air pollution.

Although antioxidant activities are increased, this does not eliminate potential health risks. If antioxidant activity is insufficient to handle the increase in free radicals and reactive oxygen species production via continuous exposure to pollutants, the result is a proinflammatory situation.^63^ For instance, it has been confirmed that ozone can initiate several inflammatory responses, such as activation of nuclear factor erythroid 2-related factor 2, heat shock protein 70, nuclear factor kappa-light-chain-enhancer of activated B cells, increased expression of a range of proinflammatory cytokines (tumor necrosis factor-alpha and interleukin 1β), chemokines, and adhesion genes.^18^,^64^ Thus we cannot eliminate acute exposure to ozone in the Ali Sabah Al Salem area.

Despite the differences in outcomes between the e-GST and total antioxidant assays, both assays confirmed the exposure of residents of Ali Sabah Al Salem to a high level of air pollution. Thus, based on the benefits of time and costs, e-GST could be used as an early biochemical marker of oxidative stress resulting from air pollution. Regarding the sampling procedure, the results suggest that finger-prick blood sampling can be considered a reliable method for evaluating blood GSTs and is a practical field method. Better patient compliance and increased patient comfort can be achieved with no need for trained phlebotomists.

### Study limitations

The limitations of the present study are mainly connected to the sampling collection. Kuwait has a conservative culture, as in other Middle Eastern countries. Therefore, no females or children participated in this study. Subsequently, all study subjects were male. Despite the presence of a professional nurse, fear of infection from an unsanitary needle lowered the rate of participation. Moreover, the concept of volunteering is extremely unpopular in the community and people are not fully aware of the importance of such research. Therefore, samples took a long time to be collected, as the team tried reaching study subjects in their homes.

The study tried to exclude as many confounding variables as possible in the selection criteria *(Supplemental Material 1),* but there was a possibility that other factors might interfere with e-GST and antioxidants other than air pollution, such as pollutants in water and food, painkillers, secondary smoke effects, occupational exposures, and genetic composition. Other variables were not considered, such as time spent living in the region, socioeconomic position, measuring airborne gases at different locations, or correlating GST activity with specific health conditions. The working conditions of the present study made it difficult to add more variables and access additional data.

## Conclusions

In conclusion, e-GST activity was lower in residents in the Ali Sabah Al Salem area compared to residents in Al-Qairawan, which suggests greater exposure to oxidative stress in Ali Sabah Al Salem. The level of pollution in Ali Sabah Al Salem exceeded the capacity of the oxidant defense system. Exposure to oxidizing agents was also confirmed by measuring total blood antioxidants, which were higher in Ali Sabah Al Salem compared to Al-Qairawan. This work emphasizes the urgent need to regulate air pollution in Ali Sabah Al Salem. The present study also confirms the use of e-GST as a biomarker for air pollution and oxidative stress in general, which can be employed for large epidemiological studies. Moreover, the present study confirms the efficacy of finger-prick blood sampling for both e-GST and total blood antioxidants. Future studies could further this work by measuring the activity of other endogenous antioxidants, enzymatic, and non-enzymatic, to assess the presence and nature of specific pollutants. Refineries and oil fields are widespread in Kuwait, and the present study could be extended to other areas to identify additional locations that pose a threat to human health.
