# Supplementary figures and images for: Effect of Air Pollution on Glutathione S-Transferase Activity and Total Antioxidant Capacity: Cross Sectional Study in Kuwait
Source: J Health Pollut. 2020 Aug 25;10(27):200906. doi: 10.5696/2156-9614-10.27.200906 (PMC7453819; doi:10.5696/2156-9614-10.27.200906)

**Supplemental Material — 1**


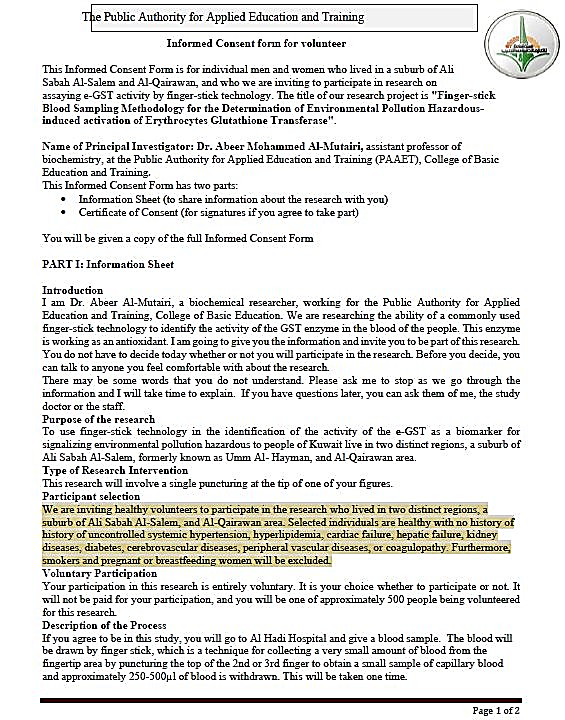


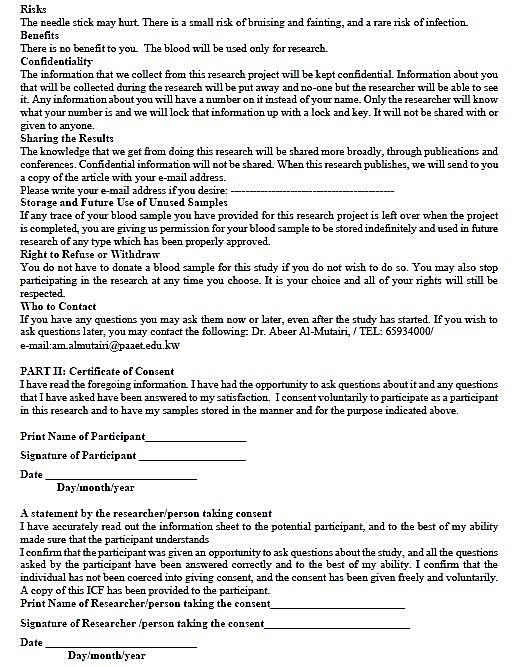

Supplement: Supplementary file 1 [file Almutairi_Supplemental1.docx]
