# Supplementary material for: Effect of Air Pollution on Glutathione S-Transferase Activity and Total Antioxidant Capacity: Cross Sectional Study in Kuwait
Source: J Health Pollut. 2020 Aug 25;10(27):200906. doi: 10.5696/2156-9614-10.27.200906 (PMC7453819; doi:10.5696/2156-9614-10.27.200906)
Supplement: Supplementary file 2 [file Almutairi_Supplemental2.docx]

**Supplemental Material — 2**

**
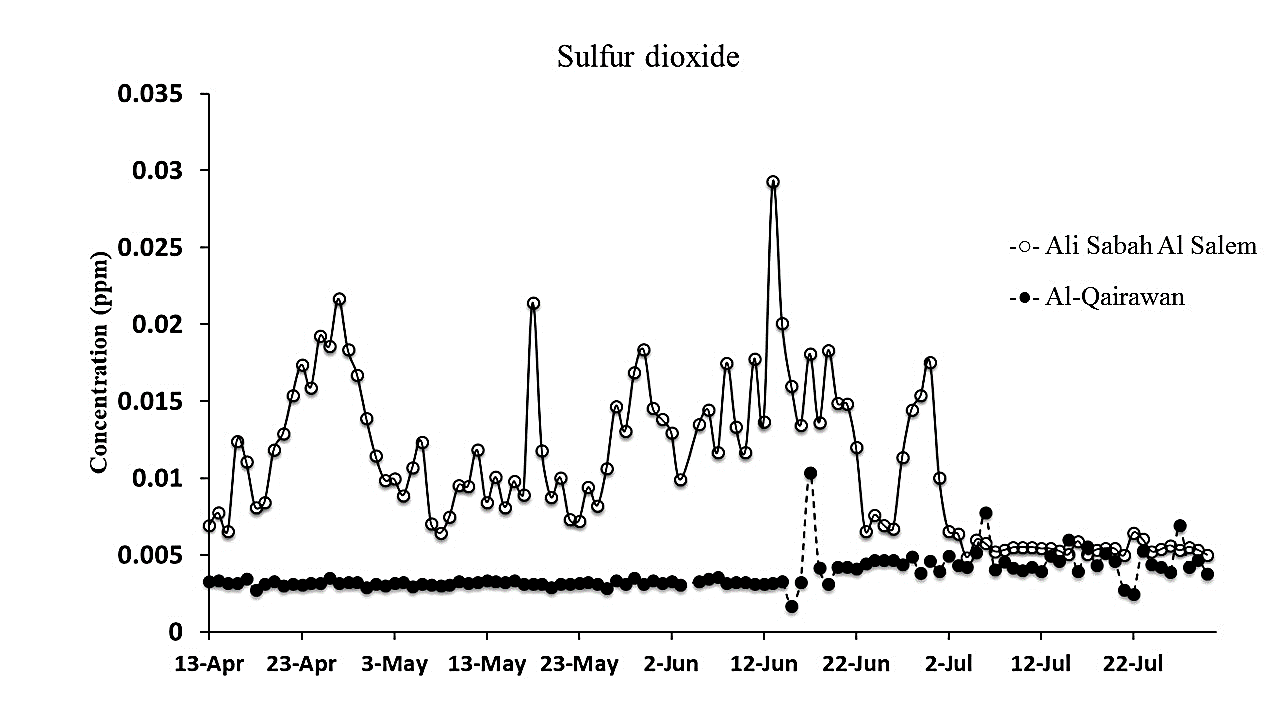
**

Figure 1: Concentrations of sulfur dioxide in Ali Sabah Al Salem, and Al-Qairawan from April 1 to August 1, 2017.

**
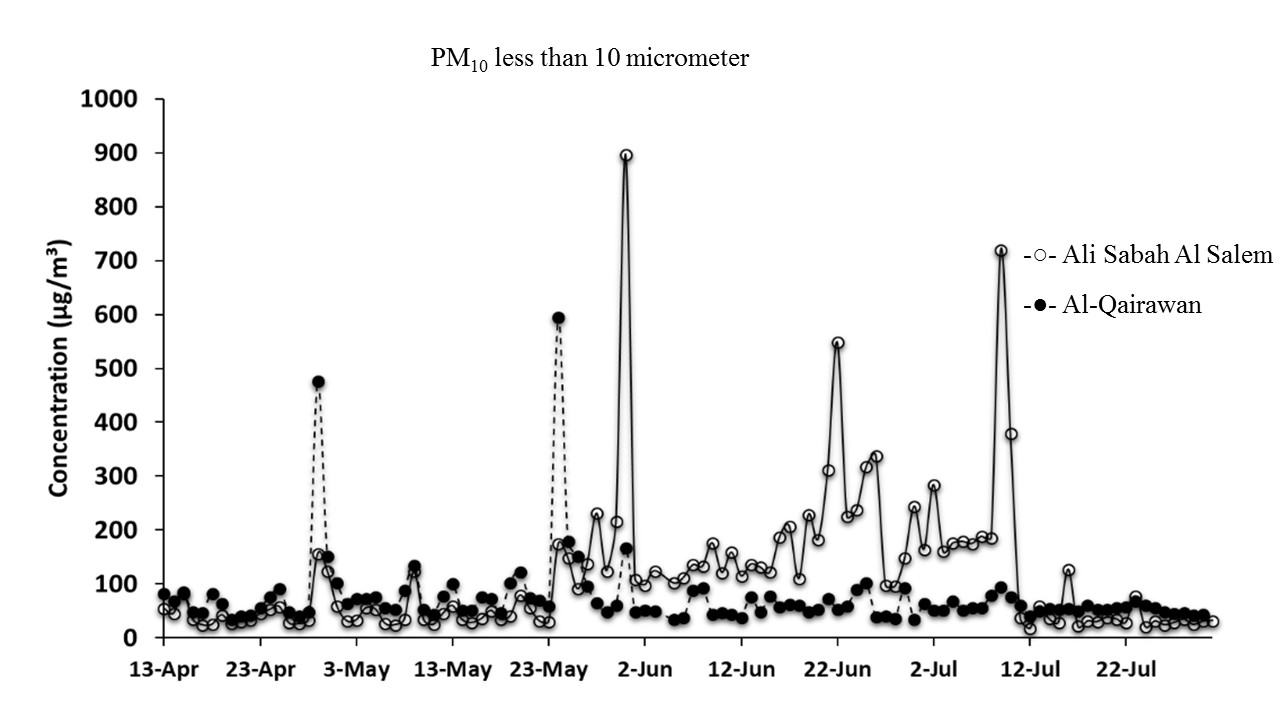
**

Figure 2: Concentrations of PM_10_ in Ali Sabah Al Salem and Al-Qairawan from April 1 to August 1, 2017.

**
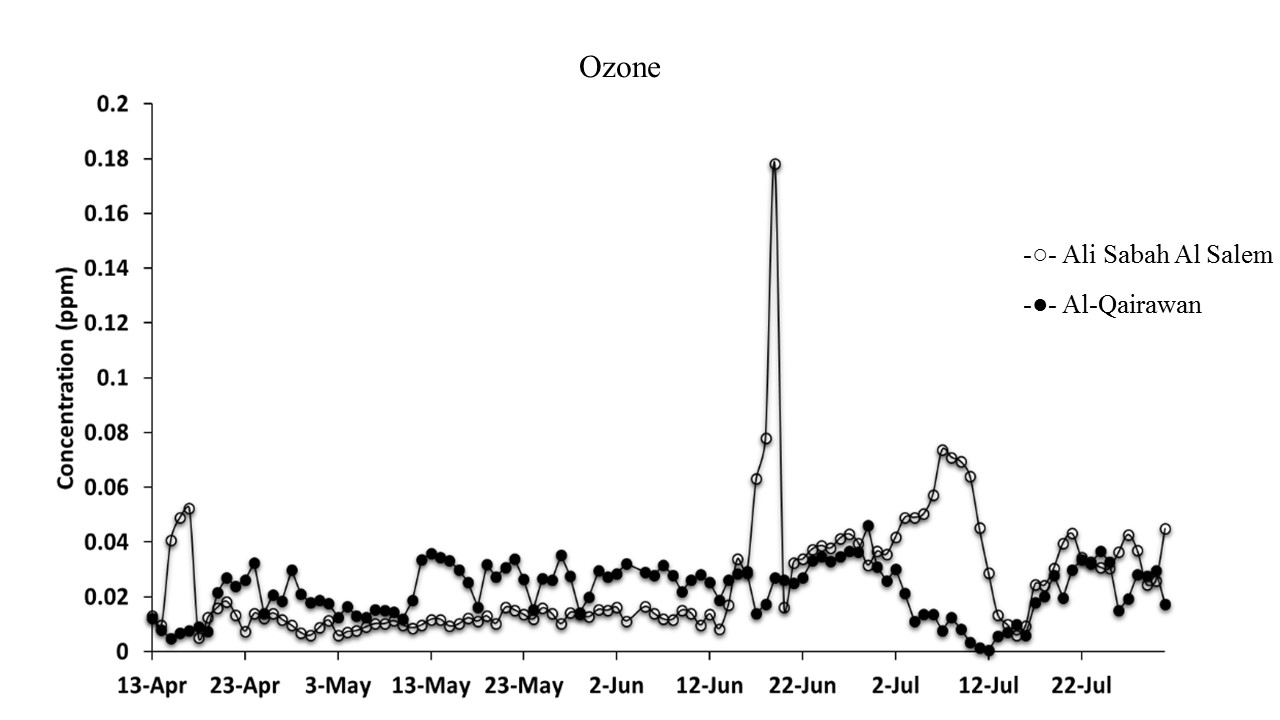
**

Figure 3: Ozone concentrations of ozone in Ali Sabah Al Salem and Al-Qairawan from April 1 to August 1, 2017.


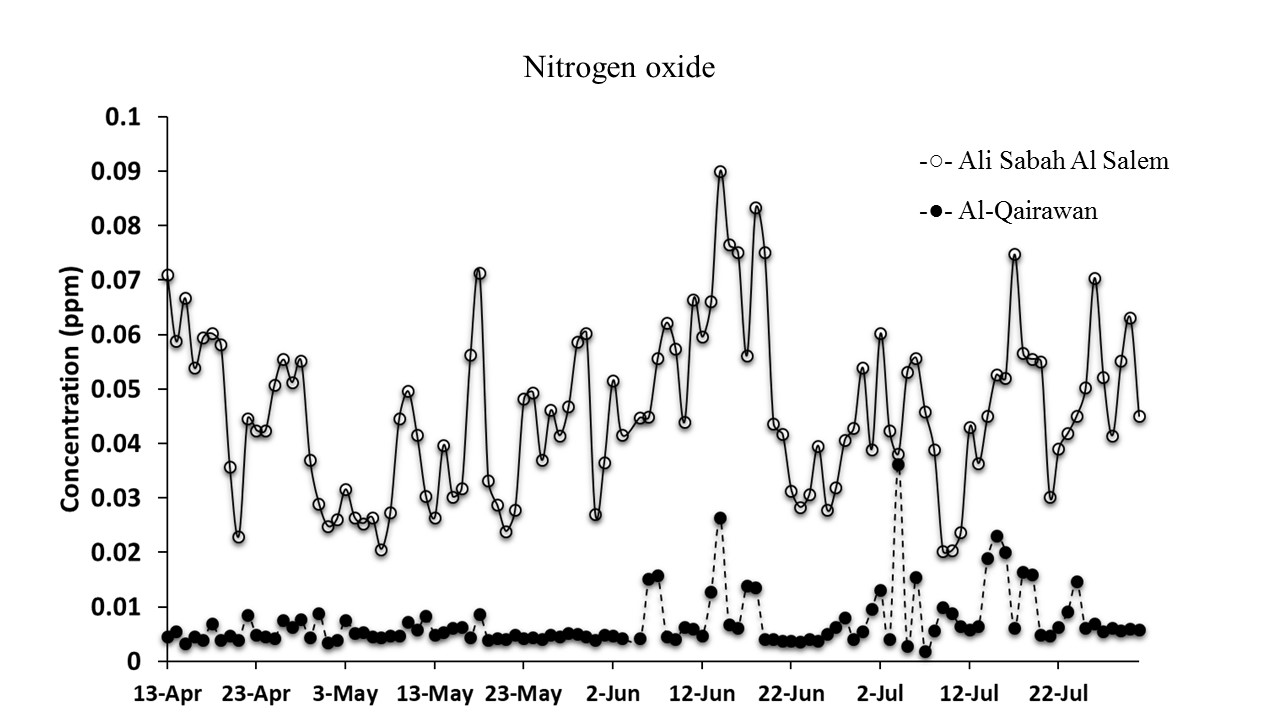


Figure 4: Nitrogen oxide concentrations in Ali Sabah Al Salem and Al-Qairawan from April 1 to August 1, 2017.

**
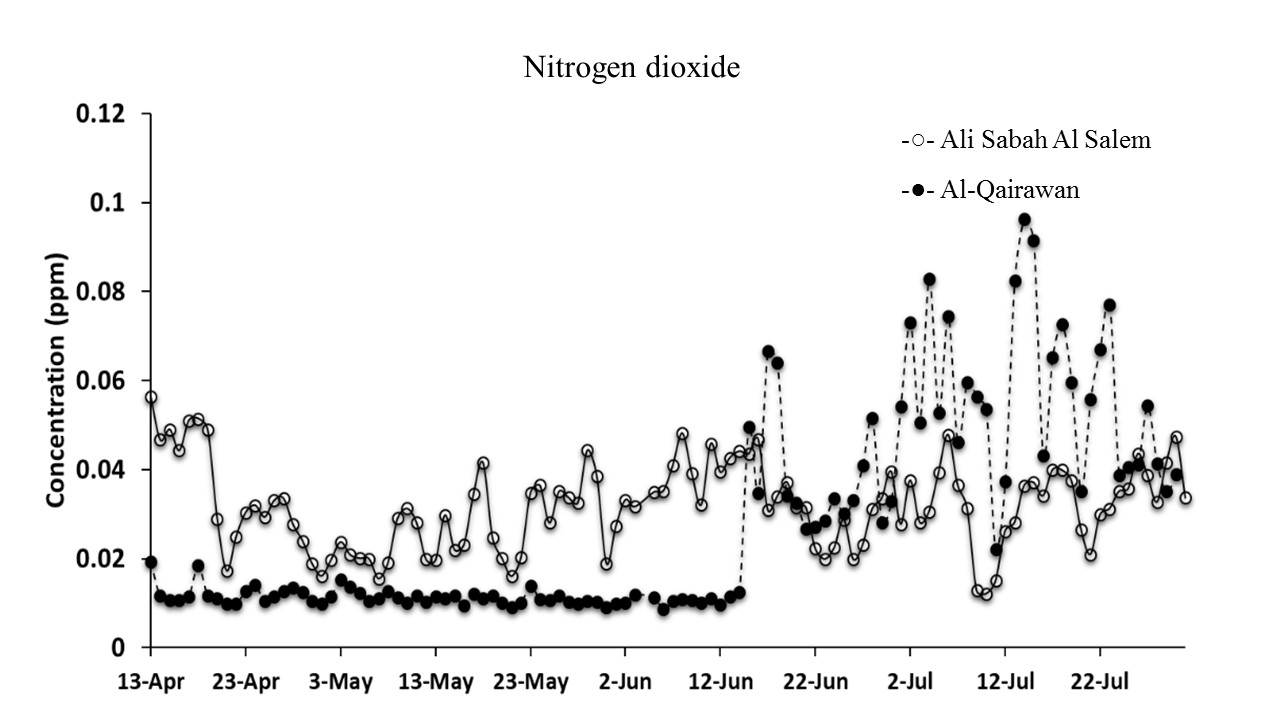
**

Figure 5: Nitrogen dioxide concentrations in Ali Sabah Al Salem and Al-Qairawan from April 1 to August 1, 2017.

**
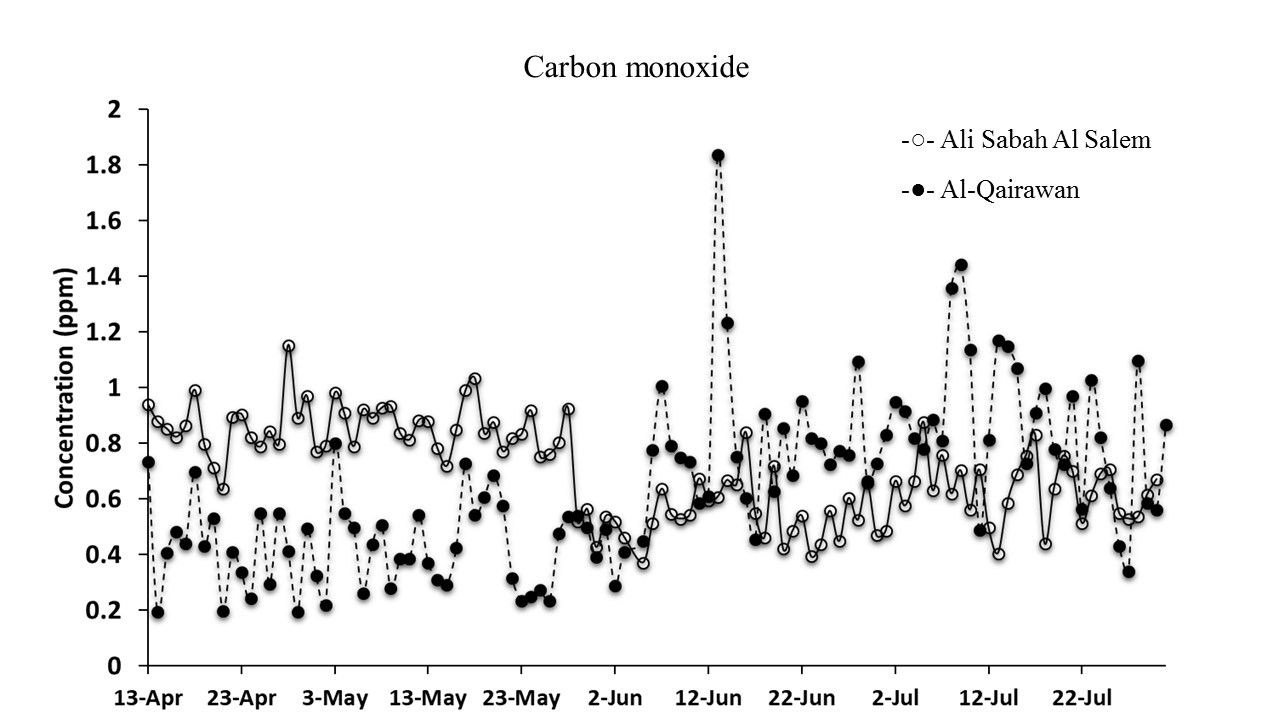
**

Figure 6: Carbon monoxide concentrations in Ali Sabah Al Salem and Al-Qairawan from April 1 to August 1, 2017.

**
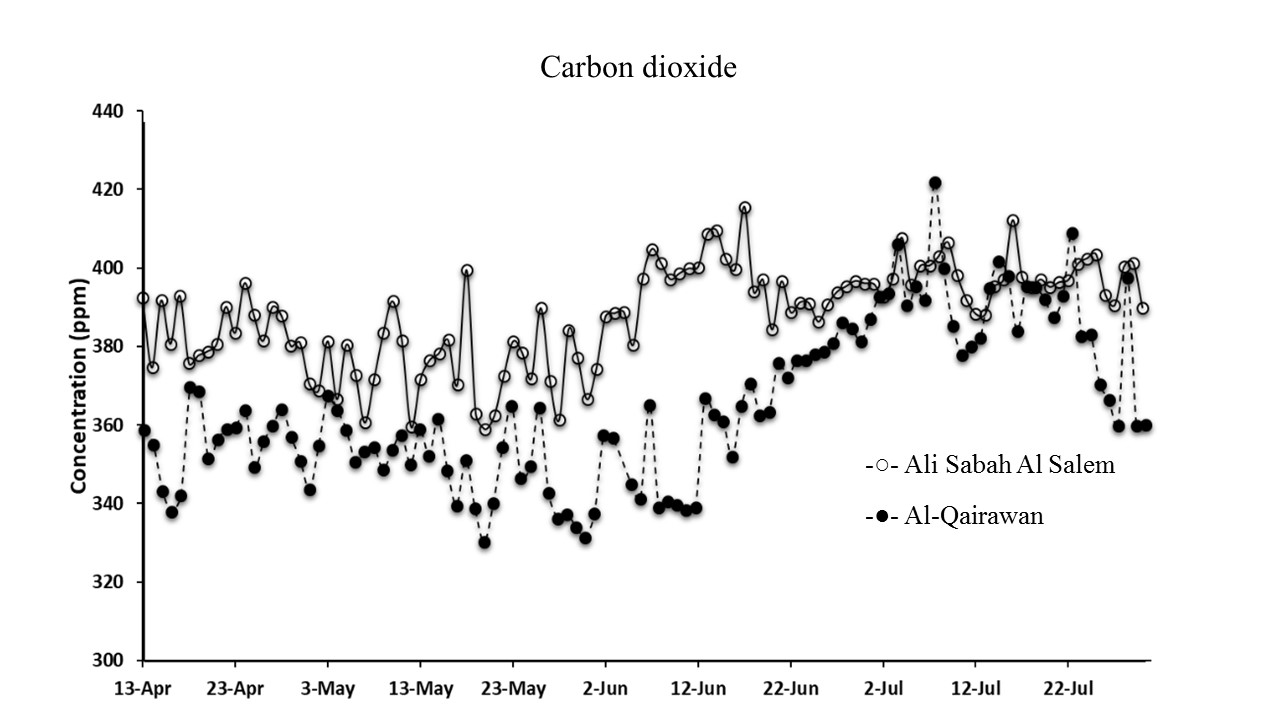
**

Figure 7: Carbon dioxide concentrations in Ali Sabah Al Salem and Al-Qairawan from April 1 to August 1, 2017.

*
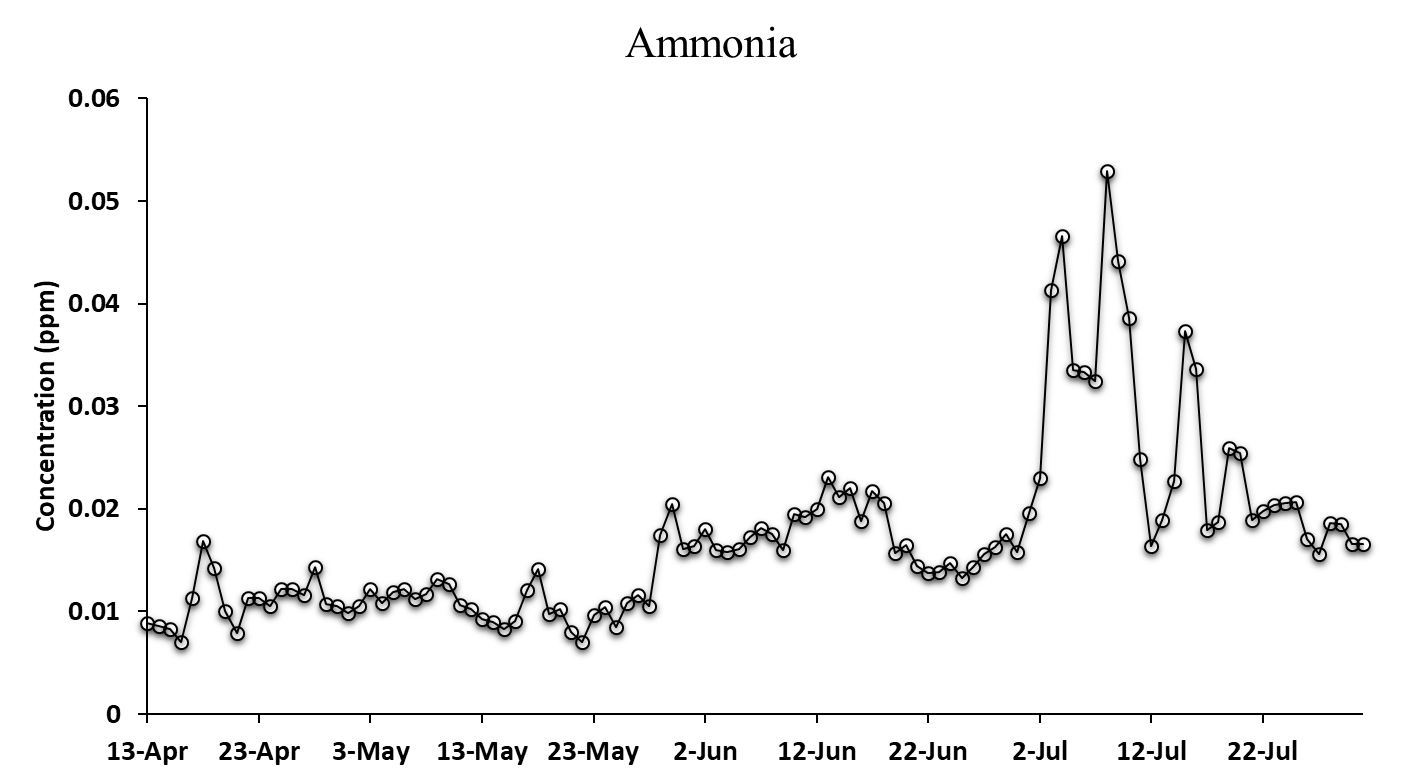
*

Figure 8: Ammonia concentrations in Ali Sabah Al Salem from April 1 to August 1, 2017.


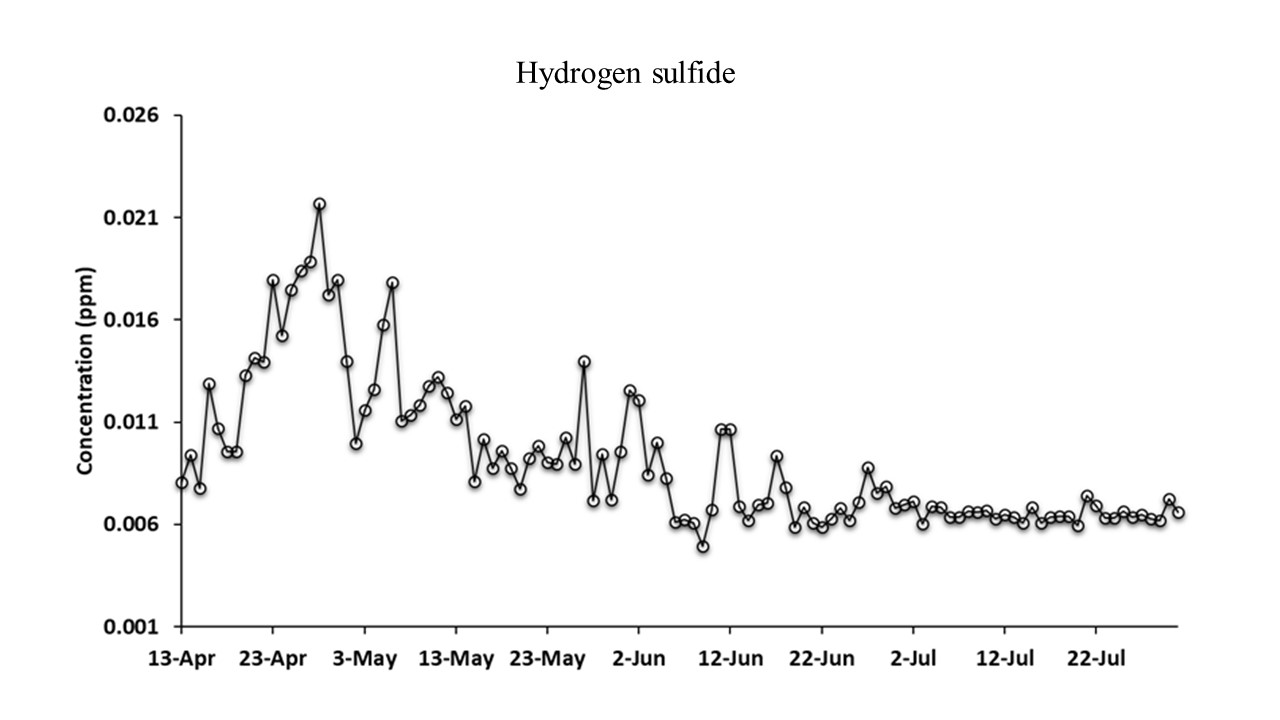


Figure 9: Hydrogen sulfide concentrations in Ali Sabah Al Salem from April 1 to August 1, 2017 .

*
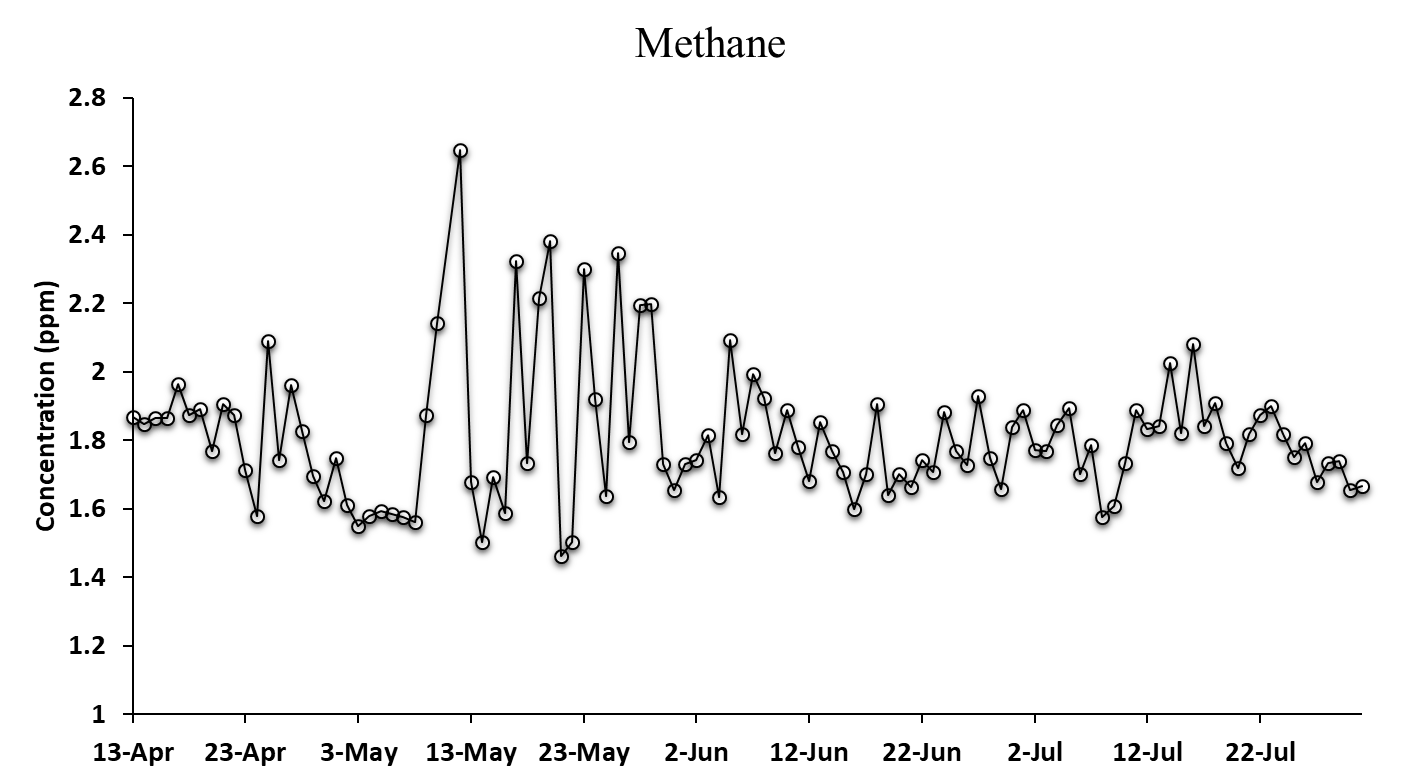
*

Figure 10: Methane concentrations in Ali Sabah Al Salem from April 1 to August 1, 2017.


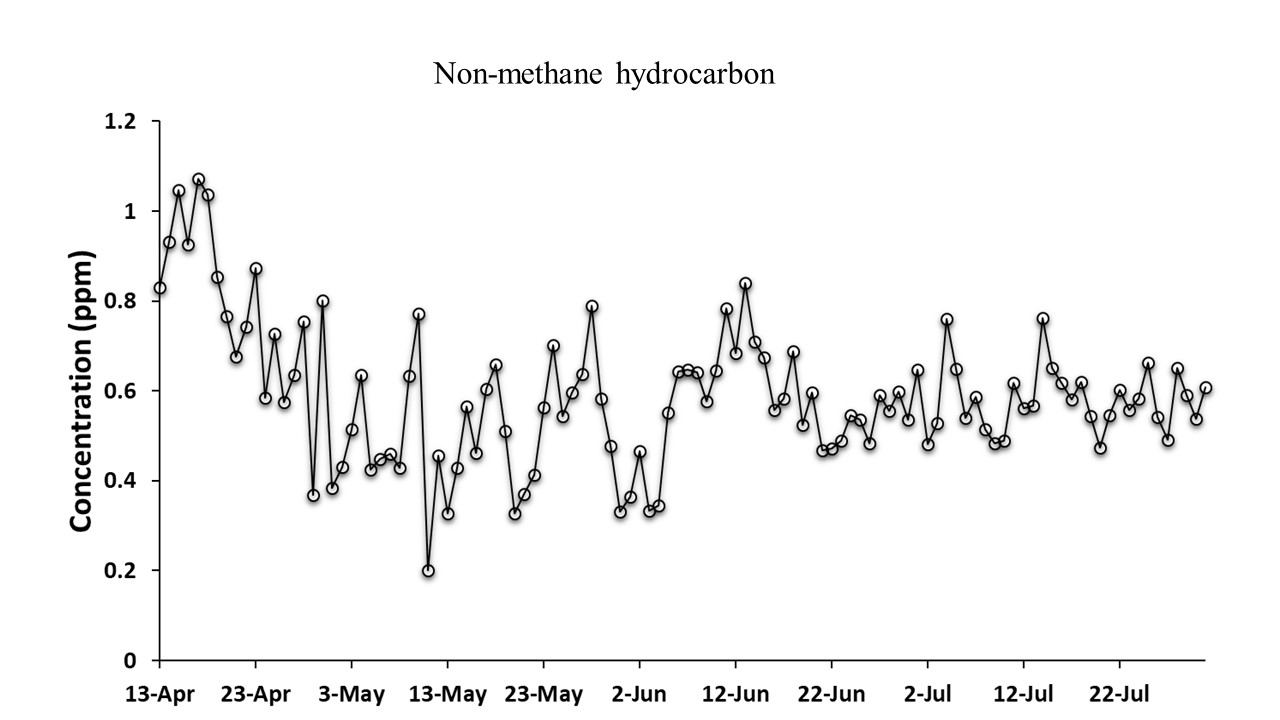


Figure 11: Non-methane hydrocarbon concentrations in Ali Sabah Al Salem from April 1 to August 1, 2017.
